# Young-onset Amyotrophic Lateral Sclerosis with Rare Skin Manifestation: Case Report and Literature Review

**DOI:** 10.7759/cureus.7844

**Published:** 2020-04-27

**Authors:** Aamna Qazi, Farheen Malik, Lubna Shafi, Saqib Basar, Azka Qazi

**Affiliations:** 1 Medicine, Dow University of Health Sciences, Karachi, PAK; 2 Internal Medicine, Dow University of Health Sciences, Karachi, PAK

**Keywords:** amyotrophic lateral sclerosis, als, young-onset als, motor neuron disease, hyperpigmentation

## Abstract

Amyotrophic lateral sclerosis (ALS) is one of the most common motor neuron diseases (MND), which presents as muscle weakness, atrophy, spasticity, and, in extreme cases, may result in death due to respiratory failure. ALS has been reported with dermatological conditions such as bullous pemphigoid and decreased collagen. Hyperpigmentation usually occurs due to underlying adrenal or metabolic disorder, but no case of hyperpigmentation has been associated with MND. We report a case of a 25-year-old man who presented with signs of young-onset ALS (progressive weakness of both upper limbs) with hyperpigmentation of limbs. The patient did not have any other underlying etiology, which could have led to the development of hyperpigmentation Biopsy was negative for polymyositis and dermatomyositis. The patient was counseled about the nature of the disease and was advised regular follow-ups.

## Introduction

Amyotrophic lateral sclerosis (ALS) is a common motor neuron disease (MND), accounting for nearly 85% of all the cases of MND. It is characterized by progressive degeneration of motor neurons of the primary motor cortex, brainstem, and spinal cord, resulting in muscle paralysis, atrophy, spasticity, and death mainly due to respiratory failure [1].

Skin manifestations like hyperpigmentation is a common symptom, but they are usually associated with endocrine diseases such as Addison's disease or hemochromatosis. Hyperpigmentation has rarely been reported with neurodegenerative diseases like ALS, Alzheimer's, and Parkinson's disease.

A thorough search in the literature revealed that although ALS has been reported with several skin conditions like bullous pemphigoid, linear cutaneous erythema, and decreased amount of collagen [2-4]. Our case is the first to report isolated hyperpigmentation alongside ALS. This makes the case very interesting as well as imperative to report as this unusual clinical presentation of an MND may confuse clinicians and cause a dilemma in diagnosing ALS. Here we present a case of a 25-year-old man with the young-onset ALS, unique in its presentation with associated localized hyperpigmentation.

## Case presentation

A 25-year-old male patient presented to the outpatient department (OPD) of Civil Hospital Karachi with a six-month history of bilaterally symmetrical weakness in both the upper limbs. The weakness was gradual in onset and was getting worse with the progression of time. In addition, he also complained of patchy hyperpigmentation over his extremities. This hyperpigmentation had also developed gradually and was black with no itching or blanching. The patient also gave the history of a solitary pustule on his back two months back. The pustule was increasing in size and discharging pus until it was treated with a course of antibiotics and healed, leaving behind a scar. He had a similar pustule on his left leg, which was treated with antibiotics, and the pustule resolved. His family history was insignificant, there was no consanguinity of parents, and he had no history of working with chemicals.

**Figure 1:**
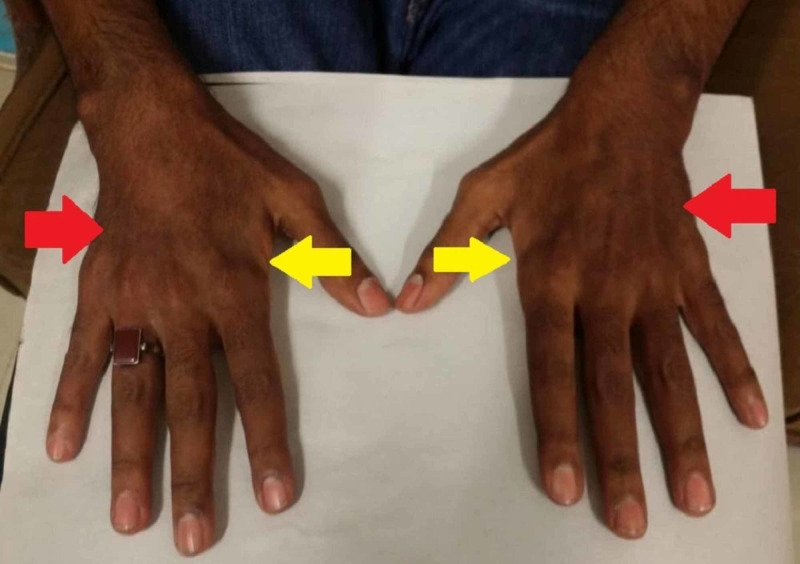
Hyperpigmentation over the dorsal surface of hands (red arrows) and guttering over first interossei (yellow arrows)

**Figure 2:**
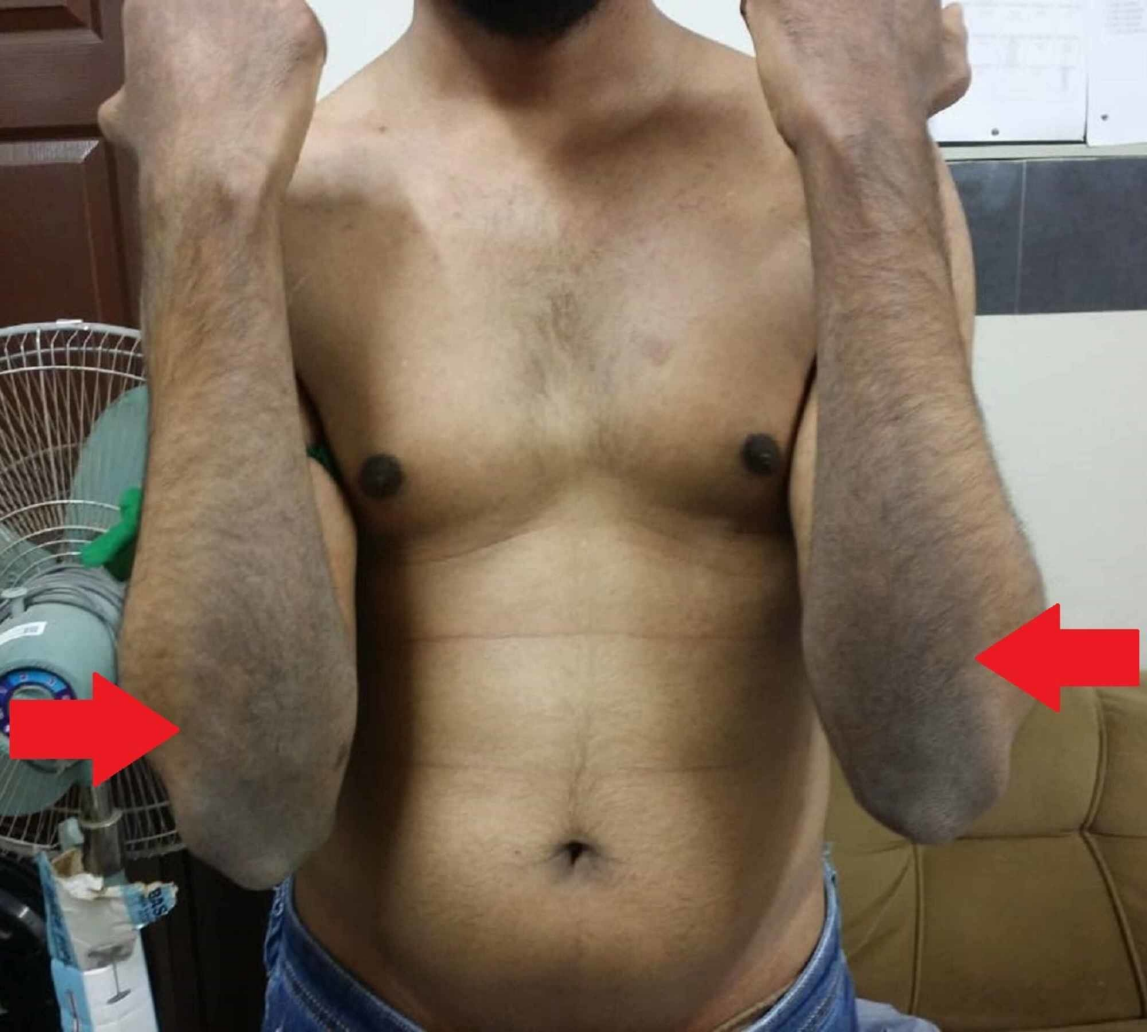
Hyperpigmentation on the extensor surface of arms (red arrows)

**Figure 3:**
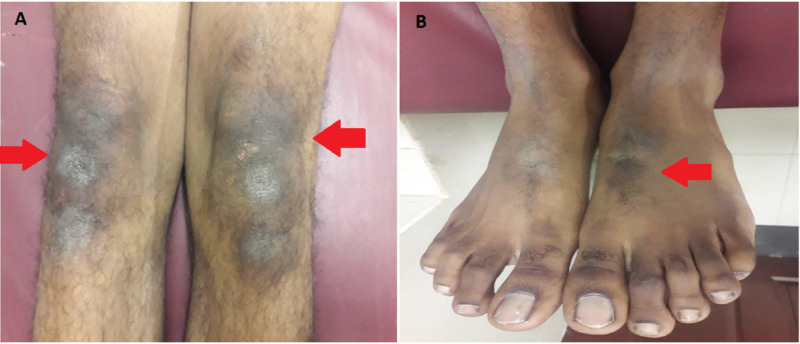
Lower limb of the patient; (A) Hyperpigmentation over knees (red arrows); (B) Hyperpigmentation over dorsal surface of foot (red arrow)

**Figure 4:**
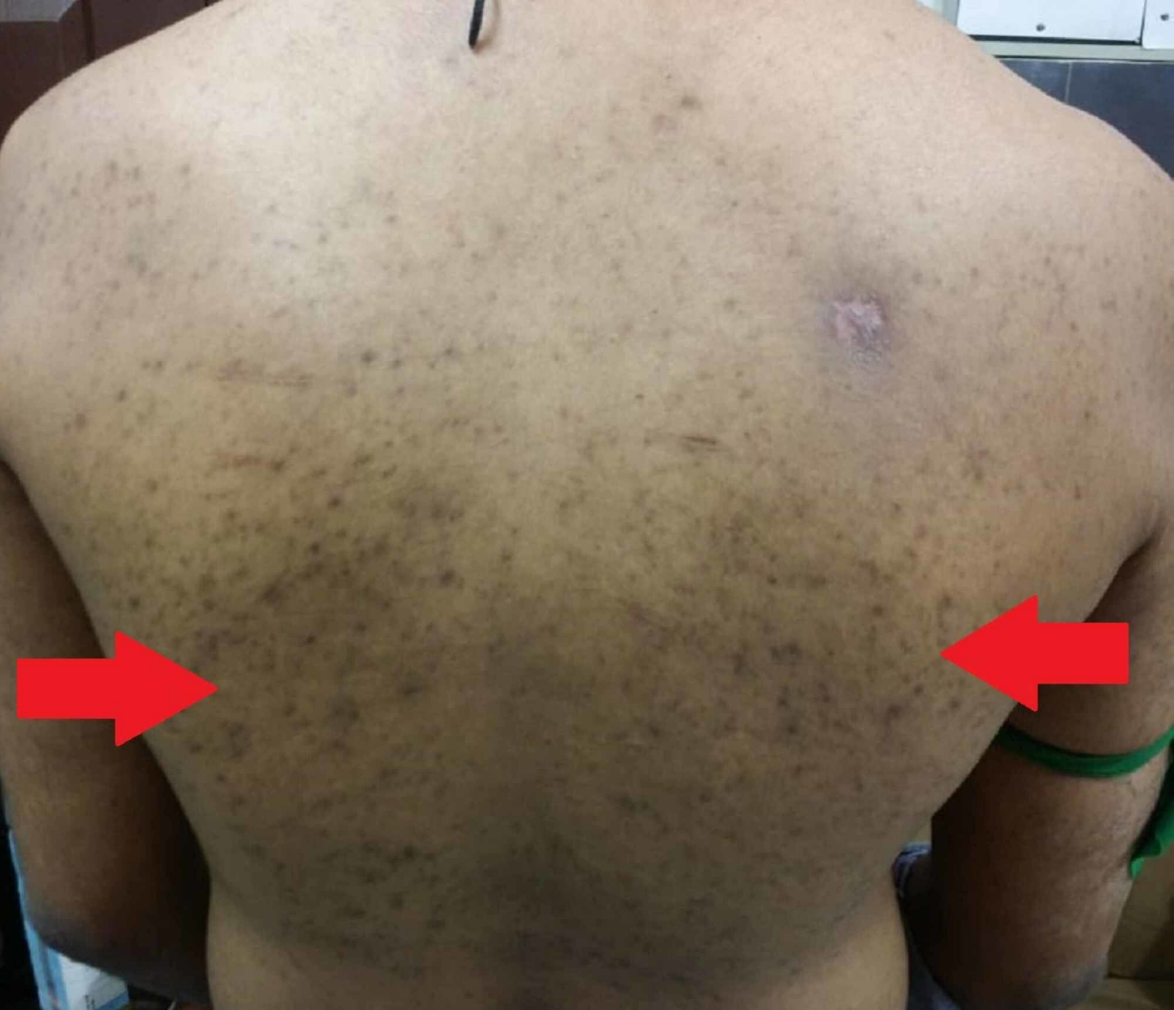
Macular rash with acneiform eruptions on the back of patient (red arrows)

On general physical examination, there were areas of hyperpigmentation over the dorsum of the hand (Figure 1), elbows, and extensor surface of arms in upper limbs (Figure 2). The lower leg also had similar areas of hyperpigmentation present on knees and dorsum of feet (Figure 3). A macular rash with acneiform eruptions was also observed on his back (Figure 4).

**Figure 5:**
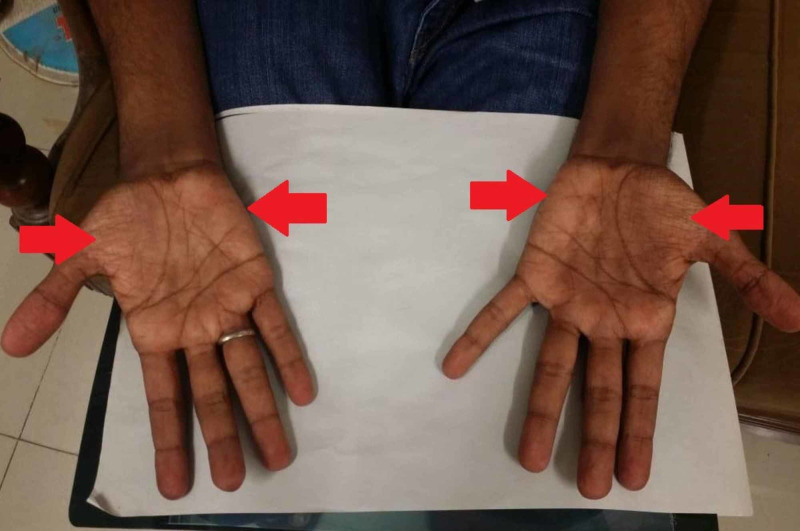
Wasting of thenar and hypothenar eminences (red arrows) on palmar surface of patient's hand

In the motor examination for the upper limb, reduction of bulk in both the deltoids, thenar and hypothenar eminences with guttering over first interossei was observed (Figure 1 and Figure 5). On examining the lower limb, a reduction of bulk in the quadriceps muscle was seen. The tone, power, and reflexes were all normal with negative Babinski sign. Mental and sensory examinations were unremarkable. Gait and speech were also normal.

Several appropriate investigations were carried out keeping dermatomyositis, connective tissue disorder, HIV/AIDS syndrome, MND, and muscular dystrophies as differentials. There were no signs and symptoms of any underlying endocrine disorders. Baseline investigations were performed, which are summarized in Table 1.

**Table 1:** Baseline investigations of the patient Hb, hemoglobin; Hct, hematocrit; MCV, mean corpuscular volume; MCH, mean corpuscular hemoglobin; MCHC, mean corpuscular hemoglobin concentration; TLC, total leukocyte count; PLT, platelet; BUN, blood urea nitrogen; Cr, creatinine; Na, sodium; K, potassium; Cl, chloride; Ca, calcium; Mg, magnesium; Phos, phosphorous; LDH, lactate dehydrogenase; T. protein, total protein; A/G ratio, albumin to globulin ratio; T. bilirubin, total bilirubin; ALT, alanine aminotransferase; ALP, alkaline phosphatase; ESR, erythrocyte sedimentation rate; FBS, fasting blood sugar

Lab Parameters	Normal Values	Results
Complete blood count		
Hb	13-18 g/dL	14.8
Hct	40%-50%	44.5
MCV	80-100 fL	83.6
MCH	27-32 pg	28.0
MCHC	31.5-34.5 g/dL	33.6
TLC	4-11×10^3^/µL	6.1
Neutrophils	40%-75%	77
Lymphocytes	20%-45%	10.9
PLT	150-450×10^3^/µL	315
Serum electrolytes		
BUN	6-20 mg/dL	20
Cr	0.7-1.2 mg/dL	0.8
Na	136-146 mEq/L	143
K	3.5-5.1 mEq/L	4.6
Cl	98-106 mEq/L	106
Ca	8.8-10.2 mg/dL	9.3
Mg	1.6-2.6 mg/dL	2.5
Phos	2.7-4.5 mg/dL	3.4
LDH	120-246 IU/L	394
Uric acid	3.4-7.0 mg/dL	4.9
Protein profile		
T. Protein	6.4-8.2 g/dL	7.1
Albumin	3.4-5.0 g/dL	4.0
Globulin	1.9-2.8 g/dL	3.1
A/G ratio	1.1-2.2	1.29
Liver function tests		
T. Bilirubin	<1.2 mg/dL	0.28
ALT	<50 U/L	68
ALP	50-136 U/L	82
Other tests		
ESR	0-20 mm/1^st^ hour	18
FBS	<100 mg/dL	84

Ultrasound abdomen was normal, and imaging did not show any mass or increase in the size of adrenal glands. Serum cortisol was also within the normal range (14.02 µg/dL, N=4.3-22.4). Tests for Hepatitis B and C virus were negative. Enzyme-linked immunosorbent assay (ELISA) and Western blot were done for detecting HIV infection, but the results were negative. Anti-nuclear antibodies (ANA) were positive. This raised suspicion of autoimmune etiology, mainly systemic lupus erythematosus (SLE), and test for the presence of anti-double-stranded DNA (anti-dsDNA) antibodies was performed, which came out negative. To explore other autoimmune antibodies, an extractable nuclear antigen (ENA) profile was done to rule out other autoimmune disorders such as systemic sclerosis, mixed connective tissue disorder, and Sjogren syndrome. The results of ENA are shown in Table 2, and all formerly mentioned autoimmune diseases were ruled out. Along the course of establishing a diagnosis, suspicion of the paraneoplastic syndrome was raised too, which was ruled out by normal levels of alpha-fetoprotein (AFP) (7 ng/mL, N<10), CA-19-9 (7.56 U/mL, N<37), and carcinoembryonic antigen (CEA) (1.78 ng/mL, N < 3).

**Table 2:** ENA profile of the patient Anti Jo-1, antihistidyl transfer ribonucleic acid synthetase; Anti-Sm, anti-Smith antibodies; Anti-SS-A (Ro), Anti-Sjögren's-syndrome-related antigen A; Anti-SS-B (La), Anti-Sjögren's-syndrome-related antigen B; RNP, ribonucleoprotein; Anti-Scl 70, anti-scleroderma 70; Anti-PM/Scl, anti-polymyositis/sclredoderma; Anti-RNP A, anti-ribonucleoprotein A; Anti-RNP C, anti-ribonucleoprotein C; Anti-RNP 70, anti-ribonucleoprotein 70; ENA, extractable nuclear antigen

Tests	Results
Anti-Mitochondrial (M2)	Negative
Anti-Jo-1	Negative
Anti-Sm	Negative
Anti-SS-A (Ro)	Negative
Anti-SS-B (La)	Negative
Anti-RNP	Negative
Anti-Centromere B	Negative
Anti-Scl-70	Negative
Anti-Ro-52	Negative
Anti-ribosomal protein	Negative
Anti-histone	Negative
Anti-PM/Scl	Negative
Anti-RNP-A	Negative
Anti-RNP-C	Negative
Anti-RNP 70	Negative

To rule out any underlying thyroid issues, thyroid function tests (TFTs), including serum thyroid-stimulating hormone (TSH) levels, were done, which were within the normal physiologic limits (1.772 mU/L, N=0.4-4.2). Liver function tests (LFTs) showed no significant finding, except ALT was slightly raised (Table 1), and renal function tests (RFTs) were normal too.

Serum creatine phosphokinase (CPK) (1436 IU/L, N=46-171) and aldolase (47 U/L, N=2.5-10) levels were raised our suspicion towards dermatomyositis. To confirm the probable differential, skin and muscle biopsy was performed. The biopsy report showed preserved fascicles with no sign of myocyte necrosis. No specific features of polymyositis or dermatomyositis were seen. Amyloid A stain was also negative.

Electromyography (EMG) and nerve conduction studies (NCS) indicated an intraspinal canal lesion affecting bulbar, cervical, thoracic, and lumbosacral segments. Active denervation was noted in most muscles. The findings were consistent with an anterior horn cell disorder. For imaging, magnetic resonance imaging (MRI) of the spinal cord was carried out but did not show any finding, and the spinal cord was normal. The patient was diagnosed with young-onset amyotrophic lateral sclerosis based on the clinical course of disease and EMG findings. The patient was counseled about the nature of the disease and advised follow up. However, we failed to label the cause of the skin manifestation found in the patient. They were a rare finding which could not be attributed to any of our differentials.

## Discussion

ALS is a fatal degenerative MND with a variable presentation. Patients predominantly present with limb-onset ALS (70%, characterized by spasticity, fasciculations, wasting, weakness, and brisk deep tendon reflexes), followed by bulbar-onset ALS ( 25%; manifested as speech abnormality and altered swallowing) [1]. Pseudo-bulbar symptoms such as emotional lability (quick and exaggerated changes in mood) and excessive yawning are also noticed in a considerable number of cases [5]. In our case, the patient presented with bilateral upper limb weakness and thus was a case of limb-onset ALS. There were no bulbar symptoms like altered speech. No changes in the emotional state of the patient were reported. Due to the young age of our patient, he was diagnosed as a case of young-onset ALS. The prognosis of limb-onset ALS in poor with a median survival of 3 to 4 years, and it is more common in males [1].

ALS decapitates an individual and is associated with many complications. The mobility of affected individuals is reduced along with difficulty in carrying out daily life activities, and the patients become bedridden [6]. The weakness of respiratory muscles leads to respiratory insufficiency, which initially manifests as orthopnea, decreased concentration, poor sleep quality, and headaches. Respiratory complications are the primary cause of death in ALS, and they include difficulty breathing and aspiration pneumonia [1,6]. Since our patient presented early, no such complications were noted; however, he had slight difficulty in daily activities like combing hair and lifting weights.

Most cases of ALS are sporadic, and only 5% of the cases are familial in origin, which is indicative of an autosomal dominant mode of inheritance [1,6,7]. Various genes are associated with the familial form of ALS with SOD1 (ALS1) being the most common. Other genes include ALS2-ALS10, ALS-FTD, and C9orf72 [6-8]. Our patient had no family history of ALS and had sporadic onset.

The pathogenesis of localized hyperpigmentation in ALS is yet to be understood. In the patient suffering from ALS, diverse patterns of changes in collagen, elastin fibers, and ground substance of the dermis can be seen. There is a rapid decrease in the number of desmosomes and isodesmosomes in the skin, which affects the cross-linking of elastin. [9]. In ALS, the skin histology shows slightly atrophic epidermis, and the edematous dermis is segregated with collagen fibrils and scarce mucinous deposits [10]. These microscopic and biochemical changes may contribute to the plethora of skin manifestations that have been described in ALS, such as the absence of bedsores, bullous pemphigoid, and linear cutaneous erythema [2,4,10].

Signs and symptoms of ALS resemble many MNDs, and the diagnosis can be puzzling. Clinically, ALS may resemble compressive myelopathy, HIV/AIDS syndrome, paraneoplastic syndrome, any tumor in spine, dermatomyositis, polymyositis, autoimmune disorders, and connective tissue disorders [1,6,11]. These were also the differentials in our case.

Diagnosis of ALS is based on clinical presentation and through a series of investigations. Baseline studies, including complete blood count (CBC), biochemistry profile, serum electrolytes, LFTs, and RFTs test, should be carried out to assess the patient’s overall health. Other specific tests include EMG, NCS, CPK levels, TFTs, autoantibody screen (ANA and ENA profile), chest X-ray, and MRI should be done in all patients who are suspected of having ALS [1,12,13]. Some investigations such as muscle biopsy, tumor markers, blood gases, viral serology, serum B12 or folate levels, genetic testing, and cerebrospinal fluid (CSF) culture should be done when needed to rule out other differentials [1]. In our patient, the majority of the investigations mentioned above were done, as stated in the case presentation. Workup for the paraneoplastic syndrome, viral diseases including HIV/AIDS syndrome, and autoimmune disease were done, and all came out negative. Imaging study (MRI) also showed no structural pathology in the spine. However, due to financial constraints and lack of family history, genetic testing was not done in our patient.

Hyperpigmentation is known to be associated with disorders such as Addison’s disease, hemochromatosis, and less commonly with graves’ disease. Addison’s disease preferentially causes hyperpigmentation of mucous membranes, oral cavity, conjunctiva, and genitalia [14]. Sun exposed areas and scars are most likely to be affected by pigmentation due to hemochromatosis [15]. Hyperpigmentation in graves diseases/hyperthyroidism is thought to be due to raised levels of ACTH and anti-TSH receptor stimulating antibody [16]. Several cases of drug-induced hyperpigmentation have also been observed. Incidences of hyperpigmentation have been associated with nonsteroidal anti-inflammatory drugs (NSAID), antimalarials, silver, zidovudine, tetracyclines, phenothiazines, and cytotoxic drugs such as cisplatin, doxorubicin, idarubicin, fluorouracil, and bleomycin [17]. For the cause of hyperpigmentation, an underlying metabolic, autoimmune, thyroid, or adrenal disorder was considered. Investigations including autoimmune workup, serum levels of TSH and cortisol, and US abdomen was done to visualize the adrenals. All these investigations were normal, along with an insignificant history of any drug use, and no cause could be attributed to hyperpigmentation.

Hyperpigmentation is known to occur in dermatomyositis (DM) and rarely in polymyositis (PM) [11,18]. Inflammatory myopathies (DM and PM) were strong differentials in our case.

DM is characterized by a heliotrope rash on upper eyelids, and erythematous rash involving the face, neck, and anterior chest (referred to as V-sign) or back and shoulders (known as shawl sign) [11]. PM is a subacute myopathy, often mimicking other myopathies and is usually a diagnosis of exclusion [19]. PM and DM are characterized by raised aldolase and creatine kinase, as were seen in our case [11]. The gold standard investigation for diagnosing inflammatory myopathies is muscle biopsy, which is characterized by inflammatory mononuclear cells infiltrate (rich in CD4+ T cells) predominantly in perimysial connective tissue and surrounding blood vessels. However, no such findings were seen in the muscle biopsy of our patient and helped to rule out DM and PM. After ruling out all the differentials with appropriate investigations discussed earlier and based on clinical presentation and supportive findings of EMG and NCS, the patient was labeled as a case of young-onset ALS.

A multidisciplinary approach is a gold standard for the management of ALS [6,20]. The management can be broadly divided into pharmacological and non-pharmacological therapy. Pharmacological therapy includes only one approved drug for the disease that is riluzole [20]. The non-pharmacological arm of management includes supportive and symptomatic treatment aimed to improve patient’s quality of life. The treatment is intended to control symptoms of depression, sleep disturbances, pain, digestive problems such as reflux and constipation. As the course of the disease progresses, the patient experiences swallowing and breathing difficulties, which lead to weight loss and respiratory insufficiency, respectively. For these advanced complications, gastrostomy and non-invasive ventilation can improve the condition and life of the patient [20].

In our case, the patient came at a very initial stage of the disease, and he was counseled about the prognosis and course of the disease. All the management options were explained to the patient in detail, and he was advised to keep on close follow-up. The hyperpigmentation in the patient was solely in association with ALS, and this is the first case ever to report such rare findings.

## Conclusions

In conclusion, this case highlights the possibility of rare manifestations of ALS. Our case presented with classical signs of MND (weakness, muscle atrophy, and fasciculations) but with associated hyperpigmentation. A thorough and detailed history was taken, and appropriate investigations were performed to rule out differentials and any other underlying autoimmune, metabolic, adrenal, or neoplastic disorders. All the tests were negative, and the hyperpigmentation could not be attributed to any other disorder. It was solely a rare manifestation of ALS. Physicians should be aware of such unusual presenting signs of ALS and not get puzzled if they see such a case.
